# Regulation of Adult Neurogenesis by Non-coding RNAs: Implications for Substance Use Disorders

**DOI:** 10.3389/fnins.2018.00849

**Published:** 2018-11-22

**Authors:** Robert J. Oliver, Chitra D. Mandyam

**Affiliations:** ^1^VA San Diego Healthcare System, San Diego, CA, United States; ^2^Department of Anesthesiology, University of California, San Diego, San Diego, CA, United States

**Keywords:** adult neurogenesis, non-coding RNA, addiction, miRNA, long non-coding RNA, endo-siRNA

## Abstract

The discovery of non-coding RNAs (ncRNAs)has been one of the central findings from early genomic sequencing studies. Not only was the presence of these genes unknown previously, it was the staggering disproportionate share of the genome that was predicted to be encoded by ncRNAs that was truly significant in genomic research. Over the years the function of various classes of these ncRNAs has been revealed. One of the first and enduring regulatory programs associated with these factors was development. In the neurosciences, the discovery of adult derived populations of dividing cells within the brain was equally substantial. The brain was hypothesized to be plastic only in its neuronal connectivity, but the discovery of the generation of new neurons was a novel mechanism of neuronal and behavioral plasticity. The process of adult neurogenesis resembles early neuronal development and has been found to share many parallels in the proper stages of specified genetic programs. Adult neurogenesis has also been found to play a role in learning and memory involved in particular hippocampal-dependent behaviors. Substance use disorders (SUDs) are an example of a behavioral condition that is associated with and possibly driven by hippocampal alterations. Our laboratory has determined that hippocampal adult neurogenesis is necessary for a rodent model of methamphetamine relapse. Due to the previous research on ncRNAs in development and in other brain regions involved in SUDs, we posit that ncRNAs may play a role in adult neurogenesis associated with this disorder. This review will cover the regulatory mechanisms of various classes of ncRNAs on the coordinated genetic program associated with adult neurogenesis with a special focus on how these programs could be dysregulated in SUDs.

## Introduction

The groundbreaking Human Genome Sequencing Consortium of 2004 determined that the species which developed the technology to undertake such an effort share much of their protein coding genome with the lowly *Caenorhabditis elegans* (International Human Genome Sequencing Consortium [IHGSC], 2004). This was very much a surprise and directly opposed to the long held concept of increasingly complex organisms requiring more complex protein-coding genomes. Though there were a similar number of protein-coding genes between these two very different species, there were large, highly conserved tracts of the genome that could be transcribed but did not encode protein. Since it was thought that RNA only served as a transmitted message to be immediately translated into protein, this led to the conclusion that these conserved but un-transcribed non-coding genes were junk DNA (Palazzo and Gregory, 2014). Follow-up RNA sequencing studies have found that most of the genome is transcribed, further implicating RNA in other integral biological roles other than a mere message to be translated into protein. In fact, it was found that nearly 80% of the human transcriptome is non-coding RNA (ncRNA; Cheng et al., 2005; Djebali et al., 2012). Since then, ncRNA research has interrogated the biological function of these molecules.

Some of the first identified functions of ncRNAs were in developmental and cancer biology (Calin et al., 2002). These functions suggest a role in proper and regulated growth, development, and cellular division. Although not particularly thought of as functions in CNS plasticity, these biological pathways have been found to play a role in the adulthood genesis and maturation of newly born neurons during a process described as “adult neurogenesis (Altman, 1962).” Much like other sites of the body with continuous cell division, the characterized niches of adult neurogenesis contain stem cells (neural stem cells; NSCs) that give rise to progenitor cells (neural progenitor cells; NPCs) and can further differentiate into neurons or glial cells (Kondo et al., 2003). This occurs in the dentate gyrus (DG) of the hippocampus and separately within the subventricular zone (SVZ) (Allen, 1912). The integration of these newly born cells can alter the function of existing circuitry within the brain.

Regulatory influence of adult neurogenesis can occur at multiple levels. First, the maintenance of the stem cell population, their dynamics of division, and survival. Second, the lineage decision and cellular fate commitment of the newly born cell derived from NSCs. Thirdly, the migration of NPCs to their final location within the brain. Finally, the proper integration of these of the newly born cells which will result in this newly born cell being nearly indistinguishable from developmentally derived cells. These stages of development can recruit various genomic regulatory mechanisms (Ma et al., 2010; Hsieh, 2012). As such, the profiling of these stages of development is mostly informed by the expression or absence of various transcription factors, structural proteins, and receptors. This coordinated expression or repression of various factors during this process can recruit ncRNA regulatory mechanisms. Thus, ncRNA regulation may occur at all of these levels (Vadodaria and Gage, 2014).

## Involvement of Adult Hippocampal Neurogenesis Within Substance Use Disorders (SUDs)

### Animal Models of Addiction

Clinically defined, addiction or substance dependence involves the loss of behavioral control over drug taking and drug seeking. The limited use of drugs with the potential for abuse is distinct from escalated drug use and the materialization of a chronic drug-dependent state. Such addiction-like behavior has been demonstrated in rodent models of intravenous drug self-administration (Koob, 1996, 2000, 2015; Koob et al., 2004). Rodents can learn arbitrary instrumental actions, such as lever pressing, to gain access to positive reinforcers, such as food or drugs of abuse (Caine et al., 1993). Therefore, intravenous drug self-administration, in which rodents are trained to self-administer drugs by pressing a lever for an intravenous drug infusion in an operant conditioning chamber, with intermittent (1 h twice a week access mimicking recreational intake in humans), limited (1 h daily access mimicking frequent, but non-dependent intake in humans), or long (extended) access (>4 h daily access mimicking compulsive intake and thus dependence in humans) has significant clinical relevance. An increase in drug availability or a history of drug intake has been shown to accelerate the development of dependence in humans. In rats, extended access to drugs of abuse, including cocaine, methamphetamine, nicotine, heroin, and alcohol, produce an escalation of drug self-administration, suggesting compulsive drug intake and therefore reflecting dependence-like behavior. Animal models of escalation, therefore, may provide a useful approach to understanding the neurobiological mechanisms responsible for the transition from limited drug use to compulsive intake and may represent a particularly suitable model for testing the hypothesis that alterations in adult brain plasticity by the drug is partially responsible for the addictive behavior.

### Animal Models of Craving

The reinstatement of drug-seeking behavior in rats is a widely used model of craving that mimics the relapse stage of addiction in human addicts (Shaham et al., 2003). Two models have been extensively used to uncover the key brain regions, brain circuitry, neurotransmitters, and neuromodulators associated with reinstatement behavior. The first is the drug self-administration paradigm. After the self-administration behavior is learned, it is extinguished by explicit non-reward; reinstatement drug-seeking behavior (e.g., lever pressing in the operant chamber) in response to a priming stimulus is then measured following a specific period of extinction. Priming stimuli include cues previously paired with drug self-administration (cue priming), acute non-contingent exposure to the drug (which is usually delivered to the rat by the experimenter; i.e., drug priming) or context (spatial location) where the drug was self-administered (context). The second is the conditioned place preference model of reinstatement, in which rats are administered the drug by the experimenter (passive exposure during training) and are tested (cue/context) in a drug-free state (Bardo and Bevins, 2000). Although both models have face validity, the self-administration model can be used to produce distinct drug intake patterns (limited vs. compulsive intake of the drug) that mimic recreational use vs. dependent use in human addicts, measure repeated operant behavior (during drug taking and seeking) that mimic an addict’s drug-response pattern, and produce high rates of relapse. Thus, the intravenous self-administration model of drug exposure appears to be best suited for studying the neural mechanisms of relapse.

### Regulation of Non-drug and Addiction-Like Behavior by Adult Neurogenesis

The precise function of adult neurogenesis in the regulation of non-drug behavior remains elusive. It is known that these neurons successfully integrate into the existing hippocampal circuitry, thus they may alter the function of this region upon maturation (Hastings and Gould, 1999; Markakis and Gage, 1999; Carlen et al., 2002). Additionally, associative learning appears to increase the generation of newly born neurons suggesting a role in learning and memory type behavior (Gould et al., 1999). It was later found that hippocampal neurogenesis seems to regulate contextual or environmental-associated learning (Shors et al., 2002). Additionally, antidepressant treatment generates higher levels of adult-born neurons (Malberg et al., 2000), suggesting that neurogenesis plays a role in behaviors associated with mood disorders (Hill et al., 2015). With more specific models of conditional ablation of adult neurogenesis, there is more research on how this process may regulate motivational aspects of behavior (Snyder et al., 2016; Karlsson et al., 2018).

**FIGURE 1:**
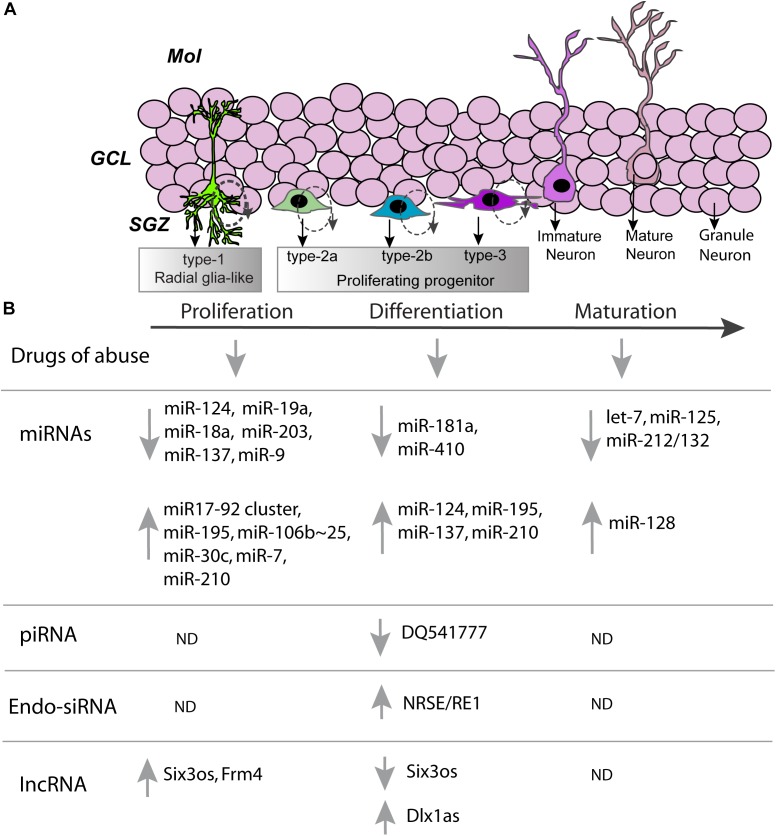
Non-coding RNAs involved in developmental stages associated with adult neurogenesis. **(A)** A cartoon representation of the dentate gyrus, with the dentate gyrus subdivided into the molecular layer (Mol), granule cell layer (GCL), and subgranular zone (SGZ). In the subgranular zone various developmental milestones of hippocampal neural stem cells are shown. Schematic of the developmental milestones demonstrating the sequence of neural stem cells (type-1), preneuronal (type-2a), early neuronal (type-2b), and post-mitotic (type-3) cell types during post-natal neurogenesis. Cells are born as type-1 radial glia-like stem cells and slowly divide to produce type-2 cells. Rapidly dividing type-2 cells differentiate into immature neuron type-3 cells and finally into a mature granule cell neuron. **(B)** Proliferation, differentiation, maturation and neurogenesis of hippocampal progenitors are altered by various drugs of abuse (see refs 28–30). Stages of neurogenesis also correlate with changes in expression of ncRNAs**:** ↑, increase; ↓, decrease; ND, not determined.

Previously, it has been found that drugs of abuse, including psychostimulants, opiates and alcohol alter the dynamics of adult neurogenesis (Figure 1; Eisch et al., 2000; Mandyam et al., 2004, 2008). This, combined with the association of adult neurogenesis with motivation and learning, suggests the involvement of adult neurogenesis in substance use disorders (SUDs) (Mandyam and Koob, 2012). Core to our laboratory’s research is the method of self-administration of psychostimulants such as methamphetamine. In this method of operant conditioning, rats are trained to associate a specific contextual environment with an action to receive drug or reinforcement, such as pressing a lever (Roberts et al., 2007). This method is the most translational addiction-like behavior model in rodents and includes many aspects of the human SUD condition, such as binge intake of a drug and propensity for relapse. In rodent research, a cognate of relapse is termed “reinstatement” and can be initiated by the drug itself, various stressors and previously drug-paired cues. For example, to test reinstatement of drug seeking-behaviors, animals may undergo a period of forced abstinence. Either immediately during abstinence or following protracted abstinence period, animals are trained to extinguish their contingent responses to drug taking as measured by lever pressing behavior in a new drug-unpaired contextual environment. In the extinction context, animals quickly learn that reinforcement is no longer associated with lever responses and therefore, reduce their lever responses. When the extinguished animal is re-exposed to previous environmental stimuli associated with drug availability, then lever responses dramatically increase signaling “reinstatement” of a previously extinguished behavior (Shaham et al., 2003). Though the dorsal hippocampus has been implicated in contextual-reinstatement for other psychostimulants, it was not known if adult hippocampal neurogenesis may regulate this form of reinstatement (Fuchs et al., 2005; Atkins et al., 2008; McGlinchey and Aston-Jones, 2018). Recent research in our laboratory has found that adult-neurogenesis during abstinence is necessary for reinstatement of drug-seeking behavior in response to re-exposure to a drug paired environment (Galinato et al., 2018). The increasing toll of SUDs on humanity has driven the need for novel interventions. Thus, the study of novel processes such as addiction-related adult neurogenesis has become increasing poignant.

### ncRNAs and Addiction-Like Behaviors

Non-coding RNAs have been found to play an important role in many aspects of addiction-related behaviors (Hollander et al., 2010; Im et al., 2010; Bastle et al., 2017). This combined with their potential function in the proper regulation of the genetic programs required for adult neurogenesis outlined below (Figure 1) suggests that ncRNAs may form an integral mechanism in the regulation of aberrant adult neurogenesis in SUDs. In the remainder of the review, we will discuss the regulatory action that may be ascribed to ncRNAs in the context of adult neurogenesis with a special focus on their potential involvement and association with SUDs.

## Classes of ncRNAs Associated With Adult Neurogenesis

### MicroRNAs (miRNAs)

The most well studied ncRNA, microRNAs (miRNAs), are often 21–22 nucleotides (nt) in length. Thus far, it has been found that miRNAs may be regulated similarly to protein-coding genes through canonical epigenetic and transcriptional pathways (Szulwach et al., 2010). They exert their regulatory function through semi-complementary binding to the 3′ untranslated regions (UTRs) of target mRNA (Tetreault and De Guire, 2013). It is this semi-complementary binding that contributes to the miRNA regulatory mechanism and sets miRNAs apart from endogenous small interfering RNAs that will be discussed later. The promiscuousness of miRNAs allows them to target wide networks of mRNAs and can induce fine tuning of these molecular networks (Guo et al., 2014). Though miRNAs initiate the targeting of specific transcripts, they do not elicit any mechanism to repress the transcript. This is done through the RNA-inducible silencing complex (RISC) which usually contains Argonaute (Ago) proteins that bind to the miRNA and have endonuclease activity. There are other mechanisms that can lead to a similar degradation of the targeted transcript or translational inhibition (Djuranovic et al., 2012). There is also some evidence of miRNA mediated regulation occurring at the 5′UTR rather than the 3′UTR but these are less studied and has not been explored in the context of adult neurogenesis (Panda et al., 2014). With this, miRNAs could play a role in many processes and have been found to be intimately involved in neuronal function in addiction-related behaviors (Bastle et al., 2018). The following section will outline the role of miRNAs within the proliferation and differentiation, migration, as well as the maturation and integration of the newly born neurons in the DG (Figure 1). When possible, specific examples will be given to point to the possibility of drugs of abuse altering these processes. Lastly, the function of miRNAs within adult neurogenesis and the impact of these regulatory molecules on addiction-like behavior will be discussed.

First proposed to be involved in mammalian tissue-specification and lineage designation, miRNAs have been associated with developmental processes for many years (Lagos-Quintana et al., 2002). The earliest, and often intertwined, stages of adult neurogenic development are proliferation and differentiation of NSCs, processes that are similar to developmentally regulated mechanisms. As a proof of concept, many initial studies would disable components of miRNA processing or machinery in the RISC. Dicer is an endonuclease that is involved in the final step of miRNA development by processing pre-miRNA to form mature miRNA to the cytoplasm (Zhang et al., 2002). Therefore, knockdown (KO) of RISC would essentially cause a blockade of all miRNA action. Researchers then developed a Dicer conditional knockout (cKO) animal to study its role in adult neurogenesis. Dicer cKO caused massive alterations in mature neuronal physiology throughout the brain, illustrating the importance of miRNAs in the baseline physiology of neurons (Cheng et al., 2014). Dicer cKO caused a reduction in the proliferative ability of NPCs in both the SVZ and the subgranular zone of the DG (SGZ). Interestingly, RISC cKO did not cause alterations in the survival of these newly born cells. However, it altered the maturation and differentiation of newly born progenitors, as cKO diminished the number of Doublecortin (Dcx)+ cells. Although this study utilized a cKO strategy to alleviate developmental effects, they initiated these studies around post-natal day 18, therefore it is hard to interpret whether or not the effects were developmentally derived issues in adult neurogenesis. In addition, the cKO was not localized to one cellular population and thus caused a systemic KO of Dicer. While there are major questions in regards to the specifics into the role or timing of Dicer and the specific miRNAs in adult neurogenesis, this study does point to the necessity for miRNAs in proper development of adult-born neurons.

Mechanisms involved in proliferation and differentiation can also be recruited during pathological conditions. For example, miR-124 was found to be downregulated by stroke/ischemia in the SVZ. Over expression (OE) of miR-124 repressed a Notch ligand, Jagged (Jag-1), which has been highly studied in embryonic development. Next, researchers found that miR-124a OE decreased NPC proliferation but promoted neuronal differentiation (Dcx+ cells), suggesting that miR-124 may shift NSCs out of a proliferating state toward a neuronal lineage (Liu et al., 2011). In contrast, stroke upregulated a particular miRNA cluster, miR17-92, within SVZ NPCs (Liu X.S. et al., 2013). OE of miR17-92 increased proliferation, whereas inhibition of a single miRNA from the miR17-92 cluster, miR-19a or 18a, suppressed proliferation and increased cell death. Addition of sonic hedgehog (Shh) protein caused a concomitant upregulation of the miR17-92 cluster along with c-Myc. To further determine the mechanism of miR17-92 regulation, they overexpressed c-Myc and found that c-Myc in the absence of Shh caused an increase in miR17-92 cluster expression. With this, the miR17-92 cluster may promote a differential mRNA network as compared to a single miRNA from the miR17-92 cluster that leads to differential effects on proliferation. Thus, pathological conditions can modulate expression of miRNAs to allow for rapid proliferation and differentiation in adult neurogenesis.

Not only do miRNAs directly regulate pathways, they may also regulate upstream regulators. For example, epigenetic regulation has been found to play a role in key processes involved in adult neurogenesis. Methyl-CpG binding protein 1 (MBD1) has been found to play a role in the proliferation and differentiation of adult neural stem and progenitor cells. Researchers determined if MBD1 may regulate the expression of miRNAs associated with either of these pathways (Liu C. et al., 2013). They found that MBD1 altered expression of many miRNAs, suggesting they may regulate proliferation or differentiation processes. Of these, miR-195 was found to form a negative feedback loop with MBD1. OE of miR-195 or MDBD1 increased or decreased proliferation and differentiation, respectively. Additionally, other miRNAs targeting epigenetic regulation have been found to play a role in proliferation. Differentiation induced downregulation of miR-203 occurs in NSCs and negatively regulates proliferation through targeting *Bmi1* mRNA which is part of a well-studied repressive epigenetic complex, the Polycomb Repressive Complex (PRC) (Liu P.P. et al., 2017). Other members of the PRC have been found to be regulated by miRNAs as well. Ezh2 is one such member that acts as a histone methyltransferase leading to inhibitory chromatin formation through histone H3K27me3. Researchers found that a miRNA, miR-137, can target Ezh2 leading to decreased genome-wide H3K27me3 (Szulwach et al., 2010). Further complicating this regulatory mechanism, miR-137 is epigenetically regulated by MeCP2 and Sox2 both of which are associated with neuronal phenotypes. Thus, they found that miR-137 OE promoted neuronal differentiation of NSCs, while KD promoted proliferation. This suggests miR-137 is associated with a non-proliferative, neuronal phenotype. Overall, these studies illustrate how miRNAs can serve as a fine-tuning mechanism to control other regulatory mechanisms involved in proliferation of adult NSCs.

As we have shown previously, drugs of abuse can alter the lineage specification of newly born cells (Yuan et al., 2011). For example, morphine appears to promote astrocytic differentiation compared to other lineages through a Notch1-dependent process (Xu et al., 2014). Researchers then set out to determine the upstream regulation of Notch1 in these cells. Other research has found that Prox1, a transcriptional repressor, can bind to *Notch1* regulatory sites and inhibit its transcription (Karalay and Jessberger, 2011). They found that *Prox1* mRNA and Prox1 association with *Notch1* regulatory sites were diminished by morphine but not fentanyl treatment. Previously, they had found that morphine and fentanyl produced differential miRNA expression in these cells. Screening these miRNAs served to identify miR-181a as a putative, yet potent, regulator of *Prox1* mRNA. Upregulation of miR-181a was found to occur through an unknown post-transcriptional mechanism, as precursor and primary forms of miR-181 were not altered by morphine. Additionally, morphine induced miR-181 upregulation mechanism appears to be Mu Opioid Receptor dependent. To determine if miR-181 could promote astrocytic rather than neuronal differentiation they transfected mouse NPC primary cultures with either miR-181 mimic or inhibitor. This led to the conclusion that miR-181 was in fact altering the differentiation dynamics and directing more NPCs toward an astrocytic fate.

Additionally, miR-124 was associated with lineage specificity and maturation. This miRNA was found to be involved in proliferation in SVZ progenitors and continued to be expressed throughout the neuronal lineage process (Cheng et al., 2009; Åkerblom et al., 2012). KO of purified NSC SVZ miR-124 caused a state continuous division, suggesting miR-124 inhibits pathways associated with proliferation (Cheng et al., 2009). On the other hand, OE of miR-124 caused increased neuronal differentiation in isolated NSCs. Similarly, in another study, diminished miR-124 expression within the SVZ led to astrocytic-like cells migrating toward and reaching the olfactory bulb (OB), the ultimate end position of SVZ derived neurons (Åkerblom et al., 2012). Cheng et al. (2009) determined that miR-124 may exert this effect through Sox9 repression, as Sox9 OE led to near blockade of neuronal differentiation and KD led to an opposite profile. Overall, this data suggests that miR-124 is mainly involved in fate determination but has some roles in proliferation of SVZ progenitor cells.

Within the SVZ, miR-410 was found to regulate a large network of genes associated with neuronal differentiation (Tsan et al., 2016). When OE in SVZ derived neurospheres, they found that miR-410 caused a preferential differentiation of astrocytic lineages while reducing neuronal and oligodendrocytic fates. However, reduction of miR-410 appeared to only promote neuronal lineages. They searched for predicted targets of the miRNA and found an RNA binding protein (RBP), Embryonic lethal abnormal vision like 4 (Elavl4) or Human Antigen D (HuD), was a strong target and associated with adult neurogenesis. RBPs can have similar mechanisms to ncRNAs, for example HuD binds to the 3′UTR like a miRNA. However, instead of translational repression and degradation of targeted mRNA, HuD promotes stabilization and translation of the transcript and can thusly upregulate the target expression. This could lead to interesting mechanisms of competition between ncRNAs and RBPs. In this case, they found that miR-410 downregulated HuD leading to differential lineage determination. Additionally, the few neurons that were produced appeared immature with small dendrites. Interestingly, HuD has been implicated in addiction-related behaviors within the striatum and may play a role in the response to drugs of abuse in other areas such as the DG or the SVZ (Oliver et al., 2018). In NSCs, researchers also found that miR-106b∼25 cluster positively regulates proliferation of these cells through an Insulin/Insulin-like growth factor-1 (IGF) mechanism (Brett et al., 2011). The miR-106b∼25 cluster may also be regulated by the Forkhead Box Protein O3 (FoxO3) transcription factor. Finally, miR-30c was found to target an axonal guidance molecule associated with proliferation in cancer, Semaphorin 3A (sema3A) (Sun et al., 2016b). OE of miR-30c was found to increase proliferation of cells in the SVZ and number of newly born neurons within the OB, suggesting that miR-30c positively regulates proliferation by inhibiting the negative effect of sema3A. Overall, the work by these researchers show that ncRNAs can play a role in determination of these newly born cells into their lineage. As well, the work by Tsan and Xu both show that this can be manipulated by exposure to drugs of abuse such as morphine (Xu et al., 2014). This aberrant lineage specification could play a role in the development of SUDs.

On the other side of proliferation is the equally regulated process of apoptosis and death of newly born cells. In the field of Parkinson’s Disease research, much focus has trained on this aspect of apoptosis and hippocampal neurogenic deficits have been suggested to be affected in Parkinson’s Disease. Cellular death in this disease has been suggested to be mediated by α-synuclein accumulation, specifically a mutant form of α-synuclein, A53T, may be especially potent. The inflammation induced by α-synuclein accumulation appears to be regulated by caspase 1 dependent mechanism within the inflammasome protein complex. Thus, researchers studied the effect of a mutant mouse containing the A53T mutation, a caspase 1 KO, or a double mutant genotype (Fan et al., 2016). First, they found that the A53T mutant presented with diminished adult neurogenesis within the SVZ and proliferation as well as differentiation of cultured NSCs. This was associated with a decrease in miR-7 expression and activated inflammasome measures *in vivo* and in cultured NSCs. They found that this was rescued in the double mutant animal, suggesting that removal of an integral inflammasome component such as caspase could increase proliferation and differentiation. Finally, they found that miR-7 targeted a component of the inflammasome, NLRP3. Using LV-OE of miR-7 within the A53T mutant SVZ, they found that miR-7 caused a normalization of adult neurogenesis in these mutant mice.

As inappropriate accumulation of a protein could cause inflammation and ultimately cell death, overexcitation of the brain can lead to similar inflammatory responses. One such regulatory mechanism is signaling from stressed mitochondria toward caspase signaling through mitochondrial-derived proteins. In seizures, researchers found that a mitochondrial protein network positively regulating apoptosis was upregulated in the DG (Schouten et al., 2015). Concomitantly, many miRNAs were upregulated including miR-124 and miR-137. This was replicated in a different laboratory through a novel Ago2 dependent sequencing technique potentially highlighting understudied RISC-loaded miRNAs following seizures (Liu X.S. et al., 2017). Though both miRNAs moderately targeted the apoptotic mitochondrial signaling protein, BCL2L13, the combinatory effect of both miRNAs was very significant. They found that this dampening of the apoptotic response was integral to preserve adult neurogenesis but allow for proper inflammatory responses to respond to the seizure. This combined with the previous study by Fan et al. (2016) illustrates that miRNAs and other ncRNAs may balance the death of non-functional adult generated neurons and a proper inflammatory response to an insult. This is a process which may be generalized to other insults such as the chronic administration of drugs of abuse, which may promote alterations in connectivity leading to addiction-related behaviors.

The proper maintenance of the NSC niche is another mechanism for appropriate proliferation. The Zebrafish pallium is a zone of adult neurogenesis. Found within this region are NSCs similar to the DG or the SVZ in mammals. During states of inactivity, NSCs may become quiescent and not participate in the generation of new cells. In the Zebrafish model, they found that these quiescent NSCs were enriched in miR-9 (Katz et al., 2016). However, the regulatory mechanism of miR-9 is very different than the canonical miRNA. Though still associated with Ago, miR-9 is mainly enriched within the nucleus as opposed to the cytoplasm where most miRNA-mediated regulation occurs. Movement of the miR-9 loaded RISC out of the nucleus appears to be associated with activation of these NSCs. Though a specific target of miR-9 was not determined, they found these nuclear localization of miR-9 was associated with Notch1 expression. They also determined that TNRC6 was the member of the RISC that allowed for nuclear localization. Although unclear, this illustrates a novel functional mechanism of miRNA induced regulation of the generation of newly born cells in adult neurogenesis.

Although much of this section has been dedicated to the precise and specific expression and regulation of miRNAs associated with adult neurogenesis, some processes in different cells have overlapping mechanisms. One such example is that of miR-210. This miRNA was found to be expressed in mature neurons, glia, and endothelial cells (Zeng et al., 2013). They found that OE of miR-210 caused a significant increase in focal microvessels within the dorsal striatum. The appearance of these microvessels may be due to the pro-proliferative effects of miR-210 on endothelial cells. Due to its role in endothelial cell proliferation when infused nearby the SVZ, they assessed the effects on proliferation and differentiation of neural progenitor cells in this region. 28 days following LV-miR-210-OE within the SVZ/DS region they detected an increase in BrdU+, Dcx+, and BrdU+/Dcx+ cells within the SVZ. However, it is unclear if this was a direct effect on NPCs, NSCs, or immature neurons within the SVZ or this was due to changes in the microvessels detected previously. Further, this highlights the interconnectivity of cellular populations within the NSC niche and the significance of miRNAs in modulating the interconnectivity to regulate the phenotype of NPCs.

The migration of newly born neurons to their proper position in the brain is an integral step of adult neurogenesis. Neurons born during development and in adulthood within the DG and the SVZ must migrate to specified positions in the brain suggesting that factors regulate the initiation and termination of newly born neuron migration. One factor that appears to play an inhibitory role on neuronal migration is miR-128 (Franzoni et al., 2015). Researchers found that immature pre-miR-128-2 was expressed in migratory immature neurons in both developmental corticogenesis and in adult neurogenic zones of the DG and SVZ. However, they found nearly non-existent colocalization of Dcx and mature miR-128 suggesting that miR-128 is downregulated during the maturation of these neurons after their final placement within the brain. Other miRNAs have been found to play similar roles in the migration of these neurons. For example, it was found that let-7 was highly enriched in migrating immature neurons from the SVZ before they reached the OB (Petri et al., 2017). When let-7 was KD, it caused improper migration of these immature neurons and retained immature morphology. In the DG, it was found that miR-19 played a similar role in the migration of newly born neurons toward the granule cell layer (GCL) and their maturation (Han et al., 2016). Thus, migration of these neurons to their proper location is sensitive to ncRNA regulatory mechanisms.

Once newly born neurons reach their defined location, the proper development of synaptic activity and integration of these cells into existing circuitry is the final stage of possibly adult neurogenesis regulation. Both developmentally derived and adult born neurons exist within the OB, as in the GCL. However, adult-born neurons may carry molecular signatures associated with the time that the cell generated. Researchers found that miR-125 may be one factor that separates these two factions in the OB (Akerblom et al., 2014). Using a novel mouse expressing GFP with a miR-125 binding site within the 3′UTR, they assessed the expression of miR-125 as indicated by lack of GFP signal. This led them to determine that adult born interneurons within the OB express miR-125 while developmentally born interneurons do not. To elucidate the function of these population differences in miR-125 expression, they used predicted targets of miR-125 to form putative pathways regulated by this miRNA. They found enrichment of pathways associated with synaptic function, thus they hypothesized that miR-125 may regulate synaptic measures. To test this, they inhibited miR-125 function by LV-mediated OE of a false miR-125 binding site mimic, or “sponge” in SVZ neurons. They found that these cells did migrate properly to the OB, but they found that these cells exhibited exaggerated dendritic complexity and length, without any effects on spine measures. When these mice were exposed to a novel odor, LV-miR-125 sponge neurons showed increased probability of Fos expression compared to GFP controls. This suggests that miR-125 negatively influences integration within the circuitry, but may slow the integration of these adult born neurons to generate proper connectivity. CREB, cAMP response element binding protein, has been found to be involved in the proper integration of newly born neurons and can regulate the expression of multiple miRNAs. For example, it was found to positively regulate miR-212/132 cluster in newly born neurons (Magill et al., 2010). KD of these miRNAs led to a decrease in dendritic length and branching, implying that miR-212/132 may target negative regulators of dendritic plasticity. This miRNA and CREB may potentially regulate adult neurogenesis that occurs during cocaine intake, as it has been found to be involved in striatal neuronal physiology during cocaine self-administration (Hollander et al., 2010; Im et al., 2010). Further, many of these miRNAs presented have played a role in the maturation of synapses and dendrites implying a role in the development of proper neuronal function within new born neurons (Sun et al., 2016a; Li and Ling, 2017).

Though this work has painstakingly shown the various mechanisms at play in adult neurogenesis that could be regulated by ncRNAs and are potentially sensitive to drugs of abuse, there has not been much discussion on the interplay of these factors leading to addiction-related behaviors or, more generally, hippocampal-dependent behavioral alterations. This will require further research on the behavioral impact of ncRNAs on adult neurogenesis and addiction-related behaviors. However, there is some evidence that ncRNAs can impact adult neurogenesis and hippocampal-dependent behaviors. Dcx is associated with an immature neuronal phenotype. It was predicted that miR-128 targeted *Dcx* mRNA and could regulate adult neurogenesis (Chen et al., 2016). With this in mind, researchers found that miR-128 OE in purified NSCs caused a decrease in neuronal lineage defined cells 16 days later. Similarly following this time course, when LV-mediated OE of miR-128 was utilized in the DG they found that 14 days later spatial learning was compromised. Spatial learning behavior analyzed was the Morris Water Maze (MWM). In this procedure, animals are placed into a large pool and must find a hidden platform to escape the water. Thus, the latency to find this platform was increased in miR-128 OE animals and could be rescued by either miR-128 KD or Dcx overexpression. Unfortunately, they did not profile the changes in the dynamics of adult neurogenesis within the brain so it is unclear if they may have caused a switch in preferential lineage determination causing MWM deficits or initiated death of these newly born cells. However, in other studies miR-128 was found to inhibit the proper migration and maturation of newly generated neurons, suggesting it may play a similar role in MWM behavior (Franzoni et al., 2015). Earlier in this review, miRNAs within the miR17-92 cluster were discussed in the role of increasing proliferation. This cluster was also found to play a role in depression-like and anxiety-like associated behaviors in mice (Jin et al., 2016). The antidepressant effect of fluoxetine on behavior is hypothesized to be due to changes in hippocampal adult neurogenesis, and has been found to dependent on miR-16 expression (Launay et al., 2011). This research shows more causality in terms of altering the dynamics of adult neurogenesis and behavior. In either case, these studies illustrate that ncRNAs can influence adult neurogenesis and concurrently alter behavior associated with this process. More work is needed to understand if drug-induced changes detailed above could alter addiction-related behaviors.

Though miRNAs by definition are negative regulators of mRNA stability, they have a diversity of downstream effects that can positively or negatively influence stages of adult neurogenesis (Figure 1). This theme applies to the other classes of ncRNAs that will be discussed. Additionally, the level of research that has been undertaken to understand the role of miRNAs within this process illuminates potential mechanisms that other ncRNAs may exert on adult neurogenesis. Drugs of abuse have also been found to regulate miRNA expression and target expression associated with adult neurogenesis. This combined with research has shown that miRNAs can play in behaviors mediated by adult neurogenesis suggest that drugs of abuse may manipulate adult neurogenesis that may potentially regulate addiction-related behavior.

### *Piwi*-Interacting RNAs (piRNAs)

The Ago family of proteins play an integral role in mediating the regulatory effects of miRNAs as established in the last section. This family can be separated into two clades of proteins containing either a PAZ domain, Ago proteins proper, or containing the namesake PIWI domain (Carmell et al., 2002). The PIWI domain is associated with a set of proteins that were discovered in *Drosophila* after searching for genes associated with germline stem cell maintenance and was named after the phenotype, P-element induced Wimpy testis (*Piwi*) (Lin and Spradling, 1997). Many years later it was found that PIWI proteins required a small guiding RNA to exert regulatory activity, similar to Ago proteins (Aravin et al., 2006; Girard et al., 2006; Grivna et al., 2006; Lau et al., 2006; Watanabe et al., 2006). These small RNAs that interact with Piwi (piRNAs) were found to be 26–30 nts and commonly containing a 5′ uracil and a 2′-*O*-methylated sugar at the 3′ most position (Vagin et al., 2006; Kirino and Mourelatos, 2007; Saito et al., 2007). Although sharing a related binding partner as miRNAs, piRNAs are a distinct class of regulatory ncRNAs.

The most well studied function and mechanism of biogenesis of piRNAs occurs through the silencing of retrotransposons. Transposons are genomic regions that can move the position in the genome. Retrotransposons (RE) are transposons that transcribed into RNA before being reverse transcribed back into DNA and inserted into the genome. Transposons can cause genomic instability, which is especially dangerous during replication of cells or in the germline. Although transposons can be destructive they are also a natural component of many genomes and have been involved in evolution and in novel regulatory mechanisms. For example, REs are most vulnerable in the RNA state. A piRNA-loaded PIWI complex may recognize a specific RE and cause endonucleolytic cleavage of the RE to prevent reverse transcription and finally re-insertion of these sequences back into the genome. In the destruction of the RE comes production of new piRNAs which can be loaded by other PIWI proteins and either silence other complementary REs or regulate other aspects of the cells (De Fazio Bartonicek et al., 2011). This unique mechanism of biogenesis for piRNAs is called “ping-pong amplification.”

One particular RE has been found to play a role in the dynamics of adult hippocampal neurogenesis. Long Interspersed Nuclear Element 1 (LINE-1) is a RE that may play a role in altering neuronal gene expression and preferential differentiation of NPCs into neurons (Muotri et al., 2005). LINE1 may also be silenced NPCs due to *Sox2* repression but *Sox2* downregulation during differentiation may lead to increased activity of this RE, indicating that somatic mosaicism of various sites of LINE1 re-insertion may be occurring within the brain. This process is tightly regulated by Wnt signaling and NeuroD1 (Kuwabara et al., 2009). Utilizing a unique L1-eGFP transgene that expresses GFP only when it is reinserted into the genome, it was found that exercise, a well-documented positive modulator of neurogenesis, increased eGFP in the mouse hippocampus (Muotri et al., 2009). Additionally, it was found that L1-insertions are enriched within the human DG suggesting this occurs naturally within human NPCs (hNPCs; Kurnosov et al., 2015). REs could also be involved in drug induced alterations in neurogenesis, such as methamphetamine in rats or cocaine in humans (Moszczynska et al., 2015; Doyle et al., 2017). Overall, this illustrates that RE such as LINE-1 may play a role in adult neurogenesis in control and addiction-related conditions. What is not known, however, is if the function of RE in this process is in fact due to the piRNAs produced from their regulation which mediates the direct effects adult neurogenesis. It does suggest that piRNAs are amplified by LINE1 expression and eventually repress LINE1, implying that this may occur in adult neurogenesis as well (De Fazio Bartonicek et al., 2011). This may be an attractive hypothesis for the function of LINE1 in adult neurogenesis.

The origin of piRNAs can be traced to other regions of the genome, such as the 3′UTR of specific genes or piRNA clusters between protein-coding genes, intergenic regions (Robine et al., 2009; Saito et al., 2009). As such, it has been found that the DG is enriched in the expression of a piRNA cluster on chromosome 17 (Lee et al., 2011). These piRNAs seem to be localized cytoplasmically, as compared to most piRNAs that are associated with nuclear functions such as silencing of REs. Not only were these piRNAs localized cytoplasmically, they were associated with dense puncta in neuronal dendrites, suggesting it may be involved in dendritic translation similar to a dendritically enriched miRNA. Focusing on one particular piRNA, DQ541777, they found that an LNA-based antisense inhibitor into cultured hippocampal neurons caused a decrease in dendritic spine area. This suggests that DQ541777 may have inhibitory effects on mRNAs that negatively influence spine dynamics. For example, they hypothesized based on sequence complementarity, that DQ541777 targeted Cdk5rap1 and Mark1/2. Although DQ541777 and its effects may be more involved in mature neuronal function, spine development is also integral to the maturation of neurons from NPCs. Additionally, they identify but did not characterize other piRNAs from this cluster that may target Astroactin, a gene associated with neuronal migration. This could play a role in immature neurons migrating through the SGZ to the GCL or in the migration of SVZ immature neurons. Although a piRNA-dependent cytoplasmic and dendritic regulatory mechanism was hypothesized by Lee et al. (2011), a direct measurement of target expression was not undertaken in this study and we are unable to conclude if these piRNAs do in fact target dendritically localized mRNAs for degradation. Thus, we are unable to conclude if they may silence genes in a similar fashion as miRNAs. In other tissues, piRNAs may play a similar role (Weick and Miska, 2014). More research is needed to understand the role that these hippocampal piRNAs may play.

Though the bulk of piRNA research has been in the reproductive organs it has been found that their associated effector complex *Piwi* proteins were found abundantly in the brain (Sharma et al., 2001). These piRNAs may play an underappreciated role in neuronal physiology. Their main function and mechanism of biogenesis in silencing of REs could be linked to hippocampal neurogenesis, especially in aberrant adult neurogenesis occurring in the context of SUD. Additionally, it appears that through a similar mechanism as miRNAs, hippocampal piRNAs may target compartmentalized mRNAs to cause alterations in structural plasticity. Further, it has been hypothesized that one of these hippocampal piRNAs targets a mRNA associated with neuronal migration and could potential regulate the proper movement of newly born neurons to move toward the GCL. Thus, piRNAs may play a role in the intersection between addiction-related behaviors and adult neurogenesis (Figure 1).

### Endogenous Small Interfering RNA (Endo-siRNAs) and Other Small Non-coding RNAs

Very little is known about endogenous siRNAs (endo-siRNAs) within mammals, let alone the mammalian CNS. For many years, it was assumed that siRNAs were an evolutionarily primitive version of miRNAs due to the mammalian interferon response to double stranded RNA (dsRNA) and were undetectable by many cloning techniques during the early discovery of miRNAs. However, it was found in *C. elegans* that a population of small ncRNAs that were highly complementary to protein-coding genes were undetected in previous studies due to modified 5′ or 3′ ends (Ambros et al., 2003; Ruby et al., 2006). Later they were discovered in mammalian cells and found to be processed by dicer but not DGCR8, the nuclease responsible for miRNA processing (Kawaji et al., 2008; Yi et al., 2009). They can be derived from Dicer processing of dsRNAs originating from the now defunct group of ncRNAs named natural antisense transcripts (Faghihi et al., 2010; Werner and Swan, 2010). These natural antisense transcripts have been found to be synaptically enriched, suggesting they may play a role in synapse specific regulatory mechanisms (Smalheiser et al., 2008). Similar to piRNAs, they can also be generated from RE silencing formed by sense-antisense dsRNA that is processed by *Dicer*. Thus, endogenous siRNAs are similar to miRNAs in their mechanism of action but are characterized by their nearly perfectly complementary to a target protein-coding sequence.

To determine if there were endo-siRNA expression patterns associated with learning, researchers trained mice in an olfactory discrimination training paradigm (Smalheiser et al., 2011a). In this paradigm, mice place their nose within one of two wells with a distinct smell in each one to nose poke for a water review. Another set of animals were exposed to the same nose-poking setup but a nose poke into either well would cause an administration of water, thus there was a lack of discriminatory learning in this pseudo-trained control group. Finally, another group was given access to the same setup without any odors thus controlling for an odorant effect. Within the hippocampus they found a large upregulation of small RNAs in the range of endo-siRNAs. These endo-siRNAs were selectively sequenced from a population that were derived from RNAse III cleavage products, due to 5′ and 3′ modifications. These endo-siRNAs were excluded if they mapped to a previously discovered miRNA. Finally, sequences derived from protein-coding gene exons or introns were considered endo-siRNAs. Thus, Smalheiser et al. (2011a) found an upregulation in these putative hippocampal endo-siRNAs that predicted behavioral consequences.

Next, they determined the genomic location of this population of learning-associated endo-siRNAs. They found that many of them were derived from plasticity-related protein coding genes such as *Abca2, Arhgef17, Camk2a, Bdnf, Gap43, Rab40b, Slc17a7, Syn1*, and *SynGap1*. Of these loci, the SynGap1 locus was enriched in many of the upregulated endo-siRNAs. From this population of endo-siRNAs, they found that one on the sense strand was predicted to form a perfect hairpin in the sense or antisense direction of Syngap1 which could result in a high probability of destruction by Dicer. Thus, we can conclude that hippocampal-dependent learning is associated with upregulation of a set of endo-siRNAs.

It is unknown if whether these endo-siRNAs are simply upregulated during learning in hippocampal neurons or if some of these endo-siRNAs may correspond with changes in adult neurogenesis within this region. It is possible that silencing of genes associated with non-neuronal lineages may be required for differentiation in adult neurogenesis. Still more, is the possibility that genes requisite for differentiation toward a neuronal fate in neurogenic processes may be lifted from endo-siRNA regulation that maintained this cell in an uncommitted state. Oddly enough, one potential endo-siRNA that positively regulates adult neurogenesis was discovered but described as a dsRNA (Kuwabara et al., 2004). This 21 nt dsRNA was found in adult hippocampal neural stem cells and both strands corresponded antisense and sense to a well-known promoter region for neuronal-specific genes recognized by the NRSF/REST transcriptional repressor. Thus, Kuwabara et al. (2004) designated the dsRNA as *NRSE/RE1*. Expression of *NRSE/RE1* appeared to increase over the development of a progenitor toward a neuronal state but not toward a glial fate, suggesting that *NRSE/RE1* is involved in neuronal-lineage determination. Overexpression of both strands of *NRSE/RE1* caused an increase in neuronal differentiation in culture as well as increased *Tuj1* promoter activity corresponding with a decrease in *Sox2, Gfap*, and *Mbp* promoter activity. Interestingly, *NRSE/RE1* did not diminish NRSF/REST expression. This implies that *NRSE/RE1* acts through a novel regulatory mechanism compared to other dsRNAs which may be processed into endo-siRNAs. This could be considered similar to a competing endogenous RNA (ceRNA) that blocks nucleotide recognition such as a miRNA (Tay et al., 2014). However, in this case it antagonizes the nucleotide binding site of a transcriptional repressor. Since endo-siRNAs are usually anti/sense toward protein-coding genes and derived from dsRNA, it follows that this may be a rare understudied mechanism for immature endo-siRNAs before they are processed into mature endo-siRNA. Thus, *NRSE/RE1* may be considered an endo-siRNA with the ability to de-repress neuronal specific genes without silencing of NRSF/REST but in competition for binding.

The mitochondrial genome is a nearly independent, yet small, genome maintained within mammalian cells including mature neurons and NPCs. Even within this compact genome, mitochondria express a small set of small RNAs termed mitosRNAs (Ro et al., 2013). Very little is known about their role in the RNA regulatory environment within the mitochondrion or possibly within the mammalian cell. However, it has been found that a group of mitosRNAs are upregulated within the hippocampus following an olfactory discrimination training procedure (Smalheiser et al., 2011b). Since mitochondrial dynamics are integral to neuronal plasticity and adult neurogenesis, these mitosRNAs may play a role in either of these processes. Overall, the role of endo-siRNAs and mitosRNAs in adult neurogenesis are promising but there is very little direct causality of these ncRNAs with adult neurogenesis or addiction-related behaviors (Figure 1).

### Long Non-coding RNAs (lncRNAs)

Characterized by their size, Long non-coding RNAs (lncRNAs) range from 200 nt up to many kilobases in length. LncRNAs are also further subdivided by their location within the genome. LncRNAs that are antisense to a particular protein-coding gene are antisense lncRNA and can be thought of as one subset of the now mostly outdated group of natural antisense transcripts that includes endogenous siRNA precursors (Faghihi et al., 2010; Werner and Swan, 2010; Sigova et al., 2013). Transcription of introns can lead to the generation of intronic lncRNA. lncRNA can be transcribed from the long stretches of DNA separating genes and are termed intragenic lncRNAs or lincRNAs. Some protein-coding mRNAs may act as lncRNAs and be transcribed, but this appears to be rare enough to not warrant recognized novel category of lncRNAs (Candeias et al., 2008; Dinger et al., 2008). Possibly due to their size compared with other ncRNAs, they have similar properties as protein-coding RNAs and are generally transcribed by RNA Polymerase II, capped, spliced, and in many cases undergo polyadenylation (Geisler et al., 2012). Overall, lncRNAs are strikingly similar to mRNA transcripts but much more diverse in the genomic origin.

As with other ncRNAs, lncRNAs form ribonucleic-protein complexes that led to their regulatory actions. Capitalizing on their length, lncRNAs can link DNA, RNA, and protein in a single complex. First detailed in the context of X chromosome inactivation (Brockdorff et al., 1992; Brown et al., 1992; Gilbert et al., 2000), the most well studied function of lncRNA is the targeting of chromatin-modifying complexes to alter the chromatin state of target loci. Through RNA-DNA interactions and or RNA-Protein interactions, they are able to direct complexes toward specific loci or obscure binding sites to prevent their activity on these loci (Mohammad et al., 2010; Schmitz et al., 2010; Wang et al., 2011; Cabianca et al., 2012; Grote et al., 2013; Johnsson et al., 2013). This can either occur adjacent to the originating lncRNA locus, targeting nearby genes in a *cis* manner, or in a *trans* manner by regulating distant loci. Similar to other ncRNAs, lncRNAs can post-transcriptionally regulate genes through altering the splicing, editing, and translational efficiency of target mRNA. Thus, lncRNAs have the most diverse range of regulatory modalities of known ncRNAs (further reviewed in Marchese et al., 2017).

Possibly owing to their high cell and tissue specificity (Cabili et al., 2011, 2015), lncRNAs have been found to play a role in early neurogenesis (Ng et al., 2012, 2013). As such, lncRNAs may play a role in adult neurogenesis as well. In an ambitious profiling study Ramos et al. (2013) utilized sequencing technologies to determine lncRNAs and protein-coding mRNAs found to be enriched in the neurogenic tissues of the SVZ, DG, and OB. One such lncRNA they identified contained intronic overlap with a neurogenic transcription factor *Pou3f3*. *2620017I09Rik* was a neighboring lncRNA found upstream of the *Pou3f3* locus. Four lncRNAs originated from four unique transcriptional start sites following the odd construction of these ncRNAs. Further, these four lncRNAs were nearby to another neurogenesis associated transcription factor *Nr2f1*. Thus, these researchers were able to identify lncRNAs specifically enriched in neurogenic tissues.

To understand the dynamics of lncRNA regulation within neurogenic tissues Ramos et al. (2013) correlated lncRNA expression with histone modifications at their corresponding transcriptional start sites. Methylation of specific lysine residues of protein-coding gene associated histones can either lead to a chromatin state that is permissive or inhibitory for transcription (Jenuwein and Allis, 2001). This study then sought to determine if lncRNA TSS H3K4me3 or inhibitory H3K27me3 were correlated with lncRNA expression similar to protein-coding genes. Many developmentally regulated genes are bivalently marked with both H3K4me3 and H3k27me3 (Bernstein et al., 2006). This is hypothesized to prime a particular gene for rapid repression or expression in a following developmental stage and, as such, may play a role in the specification of newly born neurons (Lim et al., 2009; Amador-Arjona et al., 2015). Adding to the previous knowledge that lncRNAs can be deceptively similar to mRNAs, lncRNA expression appears to be similarly regulated by chromatin dynamics with strong correlation between TSS chromatin states and lncRNA expression. Thus, they are regulated by similar chromatin dynamics as protein coding genes.

Around 928 lncRNA TSS were bivalently marked, or 10.3% of the lncRNAs expressed in the SVZ, suggesting that lncRNAs may play an important role in the differentiation and development of adult born neurons and glial cells. To further link these lncRNAs to adult neurogenesis, they hypothesized that neuronal lineage lncRNA TSSs would be bivalent in embryonic stem cells (ESCs), active in the SVZ, and inactive in non-neuronal cell types. The mRNA from these three preparations were utilized as a positive control and did in fact illustrate that three proneuronal protein-coding gene TSS, *Ascl1, Pou3f3*, and *Pou3f2*, followed this pattern (Kim et al., 2007; Hashizume et al., 2018). Ramos et al. (2013) identified 100 lncRNA TSSs that followed a similar pattern of chromatin modifications. Of these, 76% were found to be H3K4 methylated in ESCs undergoing differentiation into neurons (ESC-Neural Progenitor Cells, ESC-NPCs). Thus, even as early as the ESC-NPC stage many of these lncRNAs may be involved in regulation of the neuronal identity. The protein-coding *Pou3f2* TSS was activated which also led to an upregulation of a neighboring *lnc-pou3f2*, implying that lncRNAs originating from adjacent protein-coding genes may be coordinately regulated.

Following the hypothesis that lncRNA TSS bivalency may be important for lineage decision, it may also be involved in maintaining tissue-specific stem cells in a semi-differentiated state (Cui et al., 2009; Lien et al., 2011). Bivalent protein-coding gene TSSs present in both ESCs and NSCs were found to be associated with neurogenesis, such as *Dlx1* and *Dlx2*, validating this application to lncRNA TSS. 583 lncRNA TSSs were discovered to follow similar bivalency in both ESCs and NSCs. Thus, this population may play a role in NSC maintenance. Further, they identified lncRNA and protein-coding TSSs that are altered in a temporal fashion following differentiation and thus may play a role in regulating TSS bivalency associated with differentiation.

To more conclusively link stages of adult neurogenesis within the mouse SVZ, they performed a cell sorting experiment to separate activated NSCs (GFAP+EGFR+), transit-amplifying cells (GFAP-EGFR+), and migratory neuroblasts (CD24(+) in enriched populations away from “niche” astrocytes (GFAP(+EGFR-) and profiled lineage-specific lncRNA transcripts. This combined with all of the previously described data was compiled and sorted through to find lncRNAs TSSs which were bivalently modified in ESCs, repressed in non-neuronal cells, and were active in SVZ-NSCs^1^. They utilized *in situ* hybridization to identify an lncRNA that fit this criteria, lnc-pou3f2, was expressed in the SVZ but not the OB. Another lncRNA Six3os was found to be upregulated in SVZ-NSCs but downregulated in neuroblasts, possibly indicating a role for Six3os in the maintenance of stem-cells but not neuronal phenotypes.

Next, Ramos et al. (2013) sought to link the functionality and involvement of these differentially expressed lncRNAs in neuronal differentiation. An *in vitro* SVZ derived monolayer of cells from adult mice grown in culture is one such model to determine differentiation of this area *in vivo*. Overtime these cells within the monolayer will differentiate into glia and neurons. Within the monolayer media, researchers infused lentiviruses (LV) containing short hairpin RNA (shRNA) toward *Six3os* were utilized to knockout expression of this lncRNA. They found that Tuj1+ and Olig2+ cells were reduced with an increase in GFAP+ cells demonstrating that *Six3os* plays a role in both neuronal and oligodendrocytic lineages. They next validated *Dlx1as* which met similar criteria as *Six3os*. They found that decreased *Dlx1as* decreased Tuj+ neuronal lineage cells but increased GFAP+ cells without any changes in Olig2+ oligodendrocytic lineage cells. Further, they found that *Dlx1as* was found in the SVZ and in migratory neuroblasts within the OB. Combined, this data illustrates that the lncRNA *Dlx1as* plays a role in neuronal lineage cells but not oligodendrogenesis. To further determine the exact mechanism of lncRNA *Dlx1as*, they found that KO led to decreased neurogenic transcription factors *Dlx1* and *Dlx2*. This demonstrates that *Dlx1as* may work in *cis* to generate its effects on Dlx1/2 expression and neuronal differentiation. Thus, these lncRNAs may be involved in the *in vivo* regulation of SVZ adult neurogenesis. In conclusion, Ramos et al. (2013) completed a meticulous profiling and functional study of lncRNAs that are associated with adult neurogenesis occurring within the SVZ. These results may inform us for lncRNAs involved in adult hippocampal neurogenesis as well.

Fragile X syndrome (FXS) disrupts normal embryonic neuronal development. A mutation in the associated gene loci Fragile X Mental retardation (FMR) seems to be the originator of this syndrome. Due to FMR mutations playing a role in early neuronal development, it has been found to play a role in adult neurogenesis as well (Eadie et al., 2009; Luo et al., 2010; Guo et al., 2011; Scotto-Lomassese et al., 2011; Lazarov et al., 2012; Patzlaff et al., 2017). Though much work has been focused on the protein-coding elements of the FMR loci, it was found that a primate-specific lncRNA *Frm4* may play a role in cell proliferation and apoptosis which underlie the syndrome (Khalil et al., 2008). This lncRNA is derived from the same TSS as *Frm1* but in a reverse orientation implying that this TSS may function as a bidirectional promoter. However, they found no evidence of *cis* regulation of *Frm4* on *Frm1* in spite of their close sequence relation. Using a microarray, they found that a number of mRNA derived from distal genes were altered by Fmr4. Thus, *Fmr4* may regulate targets in *trans* rather than *cis* (Peschansky et al., 2015). This group then assessed the role of *Fmr4* in the differentiation of hNPCs given its role in proliferation. They found that *Fmr4* expression appeared to be negatively regulated by *Frm1* following 5 days of differentiation. Finally, they found that *Frm4* was largely localized with chromatin. This was in opposition to a well-studied lncRNA, *Malat1*, which participates in chromatin dynamics as well as splicing and is found nearly equally associated with chromatin or within the nucleus. Overall, this suggests that the regulatory mechanism of *Frm4* occurs through direct chromatin interaction.

To further investigate this, Peschansky et al. (2015) in their most recent study assessed the regulatory impact of OE or KD of *Frm4* on the genome-wide chromatin state. They found that nearly 430 gene promoters had differential histone methylation illustrating the various targets that may be regulated by Frm4. In OE of Fmr4, they found that 92 of these promoters had increased H3K4me3 and 86 had decreased H3K27me3 implying that these are regulated directly by *Frm4*. In contrast, in *Frm4*-KD they found that 137 promoters had decreased H3K4m3 and 115 had significantly increased H3K27me3 suggesting that these promoters are regulated indirectly by *Frm4*. Many of these genes were found to be involved in neuron-specific processes and included brain derived neurotrophic factor (*Bdnf*) and Serotonin receptor 1D (*5Ht1d*) whose mRNA was regulated accordingly to the state of the chromatin. Thus, Frm4 regulates a number of neuron-specific genes in *trans.*

Previously, Peschansky et al. (2015) found that *Frm4* plays a role in human ESC proliferation which is analogous to proliferation of NPCs in the DG. Using hippocampal (h)NPCs they sought to determine the impact of *Frm4* overexpression on the proliferation of these cells. They used ethynyl-2′-deoxyuridine (EdU) which is an exogenous mitotic marker and taken up during the synthesis phase (S-Phase) of these cells. They found that *Frm4* OE dramatically increased the number of cells with incorporated EdU, suggesting it positively influenced proliferation. Though it is mentioned that hNPCs differentiate into a heterogenous population of neurons in various states of development, astrocytes, and renewal of hNPCs after 15 days *in vitro*, it was not mentioned if *Frm4* altered the natural proportion of these differentiated cells. In either case, *Fmr4* may play a role in regulating the rate of proliferation of progenitor cells and adult neurogenesis of NPCs.

The association of lncRNAs with adult neurogenesis is established, but little is known about the direct mechanisms of many of these lncRNAs (Figure 1). The very specific *Frm4* studies by Peschansky et al. (2016) illustrate an effective model to determine their role in the proliferation of NPCs. Conversely, the work of Ramos et al. (2013) utilizes the ability of these cells *in vitro* to differentiate. This can allow for precise measurement of lncRNAs in the process of lineage determination, which is another important component of adult neurogenesis. Furthermore, many other processes integral to adult neurogenesis could be regulated by lncRNAs. One such case is the migration of these newly born cells to their proper location within the brain (Han et al., 2016). Additionally, many antisense lncRNAs have been discovered to be synaptically localized, which could imply involvement in synaptic processes integral to neuronal function in naïve and drug exposed conditions (Smalheiser et al., 2008). Nevertheless, more work is needed to determine the role lncRNAs may play in adult neurogenesis *in vivo* or in disorders such as SUDs.

## ncRNA Regulation of Substance Use Disorders (SUDs)

As with much of the research presented before, the first ncRNA to implicated as an important regulatory factor in many disease states is the miRNA. This is much the same case in addiction-related behaviors. The first miRNA to be associated with SUDs was miR-9 in the context of alcoholism (Pietrzykowski et al., 2008). Large potassium channels known as Big K+ channels (BK channels) have been associated with tolerance to alcohol. The α subunit of this channel (*Kcnma1*) is alternatively spliced leading to a variety of species of α subunits with varying sensitivities to alcohol-induced changes in BK channel function (Pietrzykowski et al., 2004). Researchers found that miR-9 targeted *Kcnma1* variants that excluded exon 29, or the Alcohol-Regulated Exon (ALCOREX), which was associated with tolerance to alcohol (Pietrzykowski et al., 2008). For most of the early foray into the effects of miRNAs in addiction, studies took a similar approach, studying the effect of a distinct miRNA-mRNA pair in cellular responses to drugs of abuse or in the context of addiction-related behaviors (Chandrasekar and Dreyer, 2009, 2011; Huang and Li, 2009; Hollander et al., 2010; Im et al., 2010; Bahi and Dreyer, 2013; Li et al., 2013; Tapocik et al., 2014; Gomez et al., 2015; Tobon et al., 2015). Eipper-Mains et al. (2011) were among the first to perform a comprehensive RNAseq to determine miRNAs altered by a cocaine regimen that produces locomotor sensitization. In this work, they characterized a wide number of miRNAs that were upregulated and downregulated within the striatum as well as utilizing bioinformatics to determine potential targets and gene networks regulated by these miRNAs. Similar RNAseq strategies were later utilized by other groups (Nunez et al., 2013; Tapocik et al., 2013, 2014; Bosch et al., 2015; Lee et al., 2015; Quinn et al., 2015; Zhu et al., 2015a; Most et al., 2016). With the proliferation of web tools dedicated to miRNA target prediction, such as TargetScan http://www.targetscan.org/vert_72/ and miRBase http://www.mirbase.org/, researchers were able to rapidly predict targets of specific miRNAs (Kozomara and Griffiths-Jones, 2014; Agarwal et al., 2015). Combined with the establishment of the Knowledge Base of Addiction-Related Genes (KARG) (Li et al., 2008), researchers were then able to search for miRNAs that preferentially targeted genes previously associated with addiction (Yan et al., 2017; Bastle et al., 2018). For example, miR-495 was identified *in silico* to target many addiction-related gene networks and was found to be associated with motivation to consume cocaine in a rat model of self-administration (Bastle et al., 2018). Overall, many miRNAs have been found to play a role in various aspects of SUD including cellular responses to drugs of abuse and addiction-related behaviors (Smith and Kenny, 2018).

Although other ncRNAs have not been studied in addiction-related contexts, lncRNAs have only recently been profiled in addiction-related behaviors, mainly as altered in response to non-contingent drug exposure (Zhu et al., 2015b). In this case, researchers profiled altered NAc lncRNA expression after mice were exposed to a locomotor sensitizing non-contingent Meth regimen. Additionally, they found evidence that these corresponding changes in NAc lncRNA might correspond with changes in addiction-associated mRNA (Zhu et al., 2016). Further research has characterized a specific lncRNA that is antisense to a synaptically localized addiction-related gene, Homer1 (Sartor et al., 2017). After repeated injections of cocaine, it was found that *Homer1-AS* was increased within the NAc. However, these lncRNAs were found to correlate with drug exposure or changes in addiction-related behaviors and not necessarily be involved in the development or response of these behaviors to drugs of abuse. It was not until a Gomafu KO mouse was developed did researchers have more mechanistic evidence for lncRNAs in addiction-related behaviors. Although much of this paper was focused on general locomotor hyperexcitability, these Gomafu KO mice were also found to be highly sensitive to the locomotor stimulating properties of Meth after a single injection. However, it is not known if Gomafu regulation of this behavior occurs in a specific brain region or even exclusively within the brain due to the systemic KO of this lncRNA. Additionally, it is not known which targets of Gomafu may be regulated to elicit increased methamphetamine-induced locomotor activity. Thus, lncRNAs are a promising yet understudied mechanism in SUDs.

## Conclusion

Non-coding RNAs represent a new and emerging field of regulation in adult neurogenesis. Throughout this review, the potential regulatory function of ncRNAs are discussed through the lens of specific pathways involved in adult neurogenesis. They are outlined as pathways involved in proliferation or differentiation of NPCs, migration, and integration of NPCs as they mature into functional neurons (Figure 1). There are many potential mechanisms for the regulation of these pathways. As discussed in this review, much of the work on this topic has been focused on miRNAs. These ncRNAs have been mainly conceived of in a negative regulatory capacity. However, their negative regulation can positively or negatively influence adult neurogenesis by regulating downstream pathways. Similarly, endo-siRNAs, piRNAs, and possibly mitosRNAs could play a similar inhibitory or “inhibitor of the inhibitor” role in processes of adult neurogenesis. On the other hand, lncRNAs appear to play a much more dynamic role in adult neurogenesis as opposed to negative inhibition of transcripts. Though the majority of ncRNAs have not been well characterized in adult neurogenesis or SUDs, miRNAs have been the best studied in this class of molecules. As such, their roles in adult neurogenesis may serve as a template for the possibilities that other ncRNAs could play. Additionally, many ncRNAs have been discovered to compartmentalize to neuron-specific regions such as the synapse, implying a very direct role in local-translation of transcripts involved in synaptic processes integral to neuronal function in naïve and drug exposed conditions. Finally, drugs of abuse have also been found to regulate ncRNA expression associated with adult neurogenesis. Research has shown that ncRNAs have been associated with behaviors mediated by adult neurogenesis or addiction-related behaviors separately. Thus, this very well may suggest that drugs of abuse could manipulate ncRNAs to alter adult neurogenesis and modulation of ncRNAs by drugs of abuse could be mechanistically linked to addiction-related behaviors.

## Author Contributions

RO wrote the review manuscript and CM and RO read the review and made final edits.

## Conflict of Interest Statement

The authors declare that the research was conducted in the absence of any commercial or financial relationships that could be construed as a potential conflict of interest.

## Footnotes

1 https://danlimlab.ucsf.edu
